# Case Report: Extrusion of a Sacral Nerve Implant Due to Engaging in Heavy Labor

**DOI:** 10.1002/ccr3.72880

**Published:** 2026-06-17

**Authors:** Yu‐Sun Guo, Wen‐Tong Ji, Xiang‐Ze Wang, Run‐Hua Tang, Jian‐Long Wang

**Affiliations:** ^1^ Beijing Hospital, National Center for Gerontology, National Clinical Research Center for Gerontology, the Key Laboratory of Geriatrics of NHC, Institute of Geriatric Medicine Chinese Academy of Medical Sciences & Peking Union Medical College Beijing China; ^2^ Fifth School of Clinical Medicine Peking University Beijing China

**Keywords:** case report, device extrusion, implantable pulse generator, sacral neuromodulation

## Abstract

Sacral neuromodulation (SNM) is an effective treatment option for patients with an overactive bladder, non‐obstructive retention, and fecal incontinence. Although the risk of device extrusion is relatively low, if it occurs, it can lead to severe complications and undermine clinical outcomes. Here, we report on a patient who experienced two episodes of device extrusion related to engaging in heavy labor. During treatments, we reimplanted the device successfully and highlight the importance of anti‐infective therapy. Specifically, salvage management included urgent debridement with high‐volume irrigation using 2 L of antibiotic solution, relocation of SNM device to a deeper subcutaneous plane (approximately 2 cm), and postoperative antibiotic therapy for 7 days. At the 7‐month follow‐up, the patient showed no evidence of recurrence. We then reviewed 9 cases related to the migration and extrusion of SNM implants, summarizing the clinical characteristics, possible triggers, and optimal treatment for clinicians when encountering similar situations.

## Graphical abstract

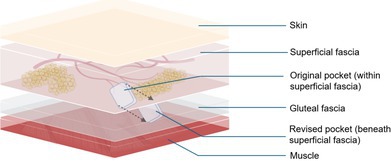

**Key Clinical Message**

In SNM extrusion, timely and anti‐infection therapy, effective debridement with high‐volume irrigation, relocation of the IPG to a deeper plane with secure fixation, and strict postoperative activity restriction may salvage the device and reduce recurrence.

## 1 Introduction

Sacral neuromodulation (SNM) is an effective treatment option for patients with overactive bladder syndrome, with or without nonobstructive urinary retention and fecal incontinence, who fail to achieve satisfactory results from conservative or medical therapies [1]. To achieve the therapeutic purpose, an implantable pulse generator (IPG) is connected to a permanent lead and placed in a subcutaneous pocket [2]. IPG‐related complications such as discomfort (10%–35%) [3, 4] and infection (2%–12%) [2, 5] are not life‐threatening, and with the improvement of surgical methods and reduction in the size of the device, discomfort caused by IPGs occurs less frequently. However, the risk of IPG extrusion, erosion, and migration requires clinical attention because it usually indicates device malfunction, inadequately formed subcutaneous pockets, and a risk of infection [6]. Given the scarcity of literature and lack of a standardized management protocol, we report a representative case of sacral nerve implant extrusion to provide additional information for clinicians. We subsequently conducted a systematic literature review and identified nine case reports related to the migration, extrusion, and erosion of SNM implants. The clinical characteristics of the patients, risk factors, and corresponding management strategies are summarized to facilitate our understanding of these complications.

## 2 Case Presentation

### 2.1 First Admission

A 53‐year‐old male farmer with a body mass index (BMI) of 24.2 was referred to the Department of Urology at Beijing Hospital in 2017. He complained of frequent urination persisting for 7 years. He was examined and received a tentative diagnosis of benign prostatic hyperplasia. The urodynamic test revealed no detrusor muscle contraction, confirming a diagnosis of a neurogenic bladder. After communicating with the patient, we planned to perform sacral neuromodulation.

One month later, the patient underwent temporary sacral nerve implantation at our hospital with good outcome. In the same month, the nerve electrode and IPG were implanted in a 1 cm depth subcutaneous pocket, within the deep layer of the superficial fascia of his right buttock. During operation, a quadripolar tined lead (Pins Medical, China; model L331) and an IPG (Pins Medical, China; model G131) were used and after lead insertion, the tines were expanded posteriorly in an outward orientation to resist outward displacement. The patient was in good condition at discharge, and a follow‐up visit 1 month later revealed satisfactory improvement in urination symptoms.

### 2.2 Readmission

**FIGURE 1:**
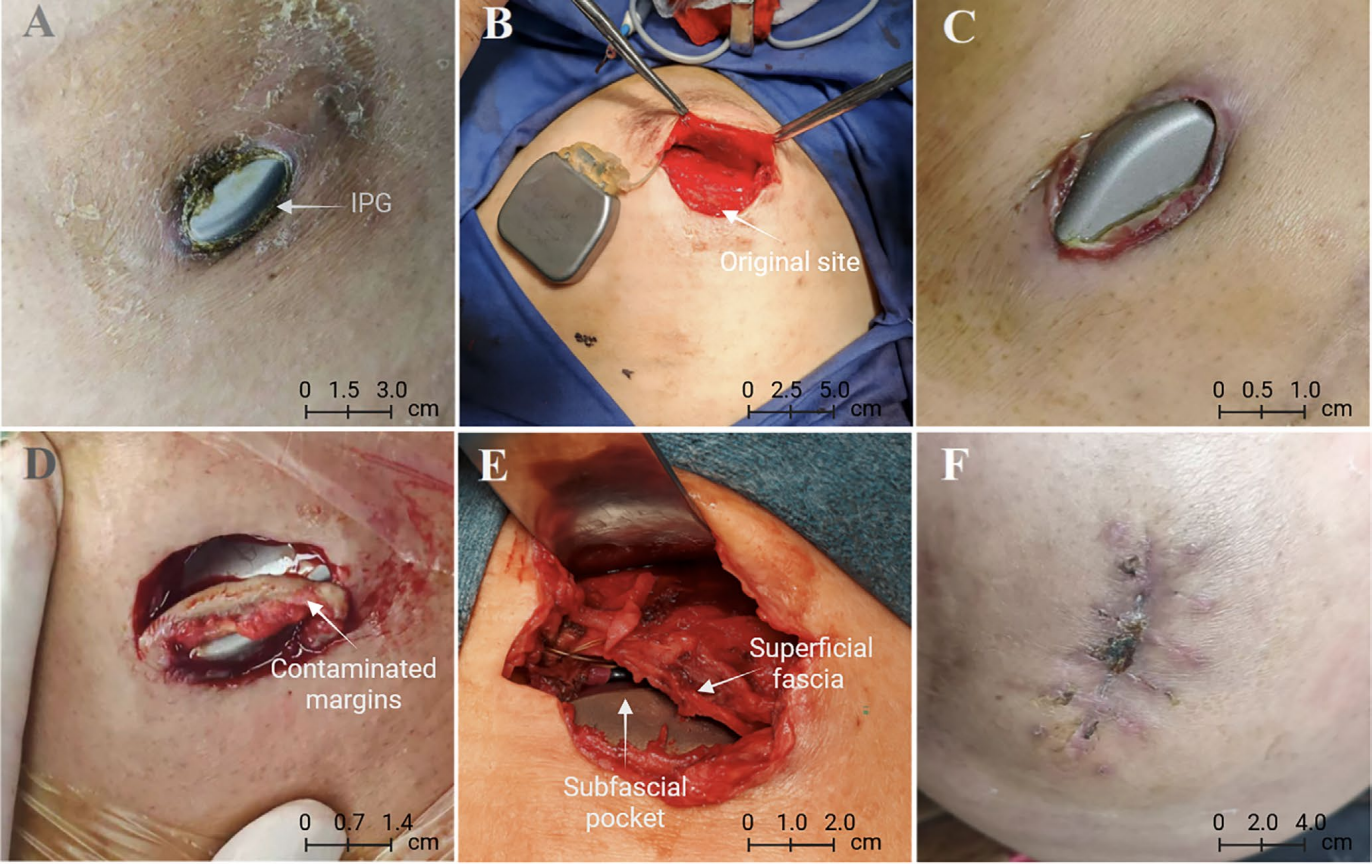
Images of the wound at different timepoints. (A) The wound at readmission. (B) The original subcutaneous pocket was dilated during the operation. (C) The wound at the third admission. (D) Removing the contaminated skin margins during operation. (E) Constructing a deeper pocket to place the device. (F) The wound at follow‐up after third admission.

82 months (6 years and 10 months) later, the patient complained of intermittent dull pain at the site where the IPG was implanted and a palpable protruding mass for 2 weeks. Upon physical examination, we found a 3 × 1.5 cm wound at the original implantation site, through which the device was extruding. No signs of infection such as pus or redness were observed (Figure 1A) and laboratory findings were within normal ranges, supporting the absence of active infection. The patient was a farmer whose work involved repetitive bending and squatting. From a biomechanical perspective, such repetitive labor is likely to increase shear stress and friction at the interface between IPG and tissue, as well as tension over the surgical incision, particularly in the current case with limited soft tissue coverage (BMI 24.2). In this context, we deduced that the prolonged and repetitive physical labor may represent a potential contributing factor to device extrusion.

Based on the intact function of the IPG and the patient's desire to continue neuromodulation therapy, re‐implantation of the device was planned. After active preoperative anti‐infective treatment, we dilated the subcutaneous pocket and re‐implanted the device at its original site within superficial fascia (Figure 1B). During surgery, we performed an adapted irrigation procedure in the surgical field to minimize the risk of postoperative infection. The irrigation solutions were used sequentially as follows: (1) one‐half strength hydrogen peroxide (500 mL), (2) one‐half strength iodophor solution (250 mL), and (3) 2 L of antibiotic solution comprising 1 g of vancomycin hydrochloride and 0.4 g of amikacin sulfate. Each solution was used with brief contact with the tissue before suction and subsequent irrigation. Vancomycin hydrochloride was intravenously transfused during and after surgery at a dose of 15 mg/kg every 12 h. Postoperatively, the surgical wound was managed with routine sterile dressings, which were changed every other day. Negative‐pressure wound therapy was not used, because there was no extensive tissue necrosis or uncontrolled infection. Upon discharge, the patient was prescribed oral levofloxacin 500 mg once daily and cefaclor 250 mg every 8 h for 1 week and advised to avoid heavy physical labor.

### 2.3 Third Admission

The patient's IPG was extruded again 16 months after readmission. A 1.5 × 1.5 cm wound at the IPG site was noted and was red, swollen, and tender, with pale white pus present (Figure 1C). Bacterial culture was consequently obtained from the wound at the site of skin extrusion prior to perioperative antibiotic therapy. Examination results indicated that the patient had no fever, normal laboratory inflammatory markers, and negative bacterial cultures. Upon inquiry, we learned that he had not followed our advice and continued to engage in heavy physical labor, including bending and squatting. We created a second surgical plan for the patient. Through preoperative discussion, the possibility of biofilm formation was considered; however, no clinical or microbiological evidence of active infection was identified to support the possibility of biofilm‐associated infection. Therefore, a decision was made to reimplant the SNM system.

**FIGURE 2:**
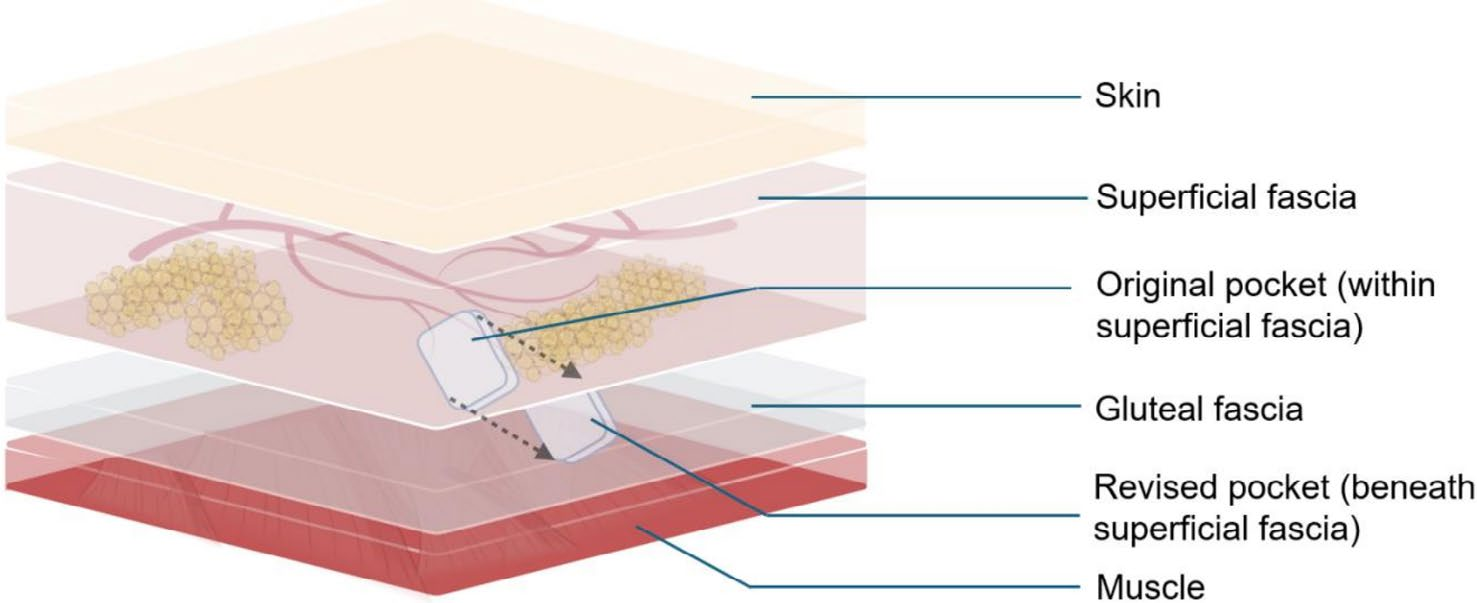
Illustration of the anatomical tissue layers and implantable pulse generator pocket planes.

In standard SNM implantation, the IPG is usually positioned within a subcutaneous pocket in the lateral–superior quadrant of the buttock, created by blunt dissection beneath Scarpa's fascia (superficial fascia). The pocket is typically of sufficient depth to accommodate the device and allow lead coiling [7]. In our case, after opening the superficial fascia at the original implantation site, we first performed circumferential excision to remove the contaminated skin margins (Figure 1D). Subsequently, blunt dissection was performed to create an approximately 2 cm–deep pocket beneath the superficial fascia and above the gluteal fascia, into which the device was placed (Figure 1E). To further clarify the anatomical relationship and the change of pocket plane, a schematic illustration is provided (Figure 2). Considering the patient's risk of infection, we adjusted the anti‐infection regimen. During the operation, a mixed solution was prepared by dissolving 0.5 g vancomycin hydrochloride, 2.25 g piperacillin‐tazobactam, and 5 mg of amphotericin B5 in 2 L of sterile water and was used to repeatedly wash the wound following the irrigation of half‐strength hydrogen peroxide (500 mL) and iodophor solutions (250 mL), each applied with brief tissue contact before suction. During and after the operation, intravenous vancomycin at 15 mg/kg per dose every 12 h was administered. The postoperative wound‐care protocol followed the same methods as described during readmission. Upon discharge, oral levofloxacin (500 mg once daily) and cefaclor (250 mg three times daily) were prescribed for 1 week. The surgery was successful, and the patient had a good early functional recovery.

**FIGURE 3:**
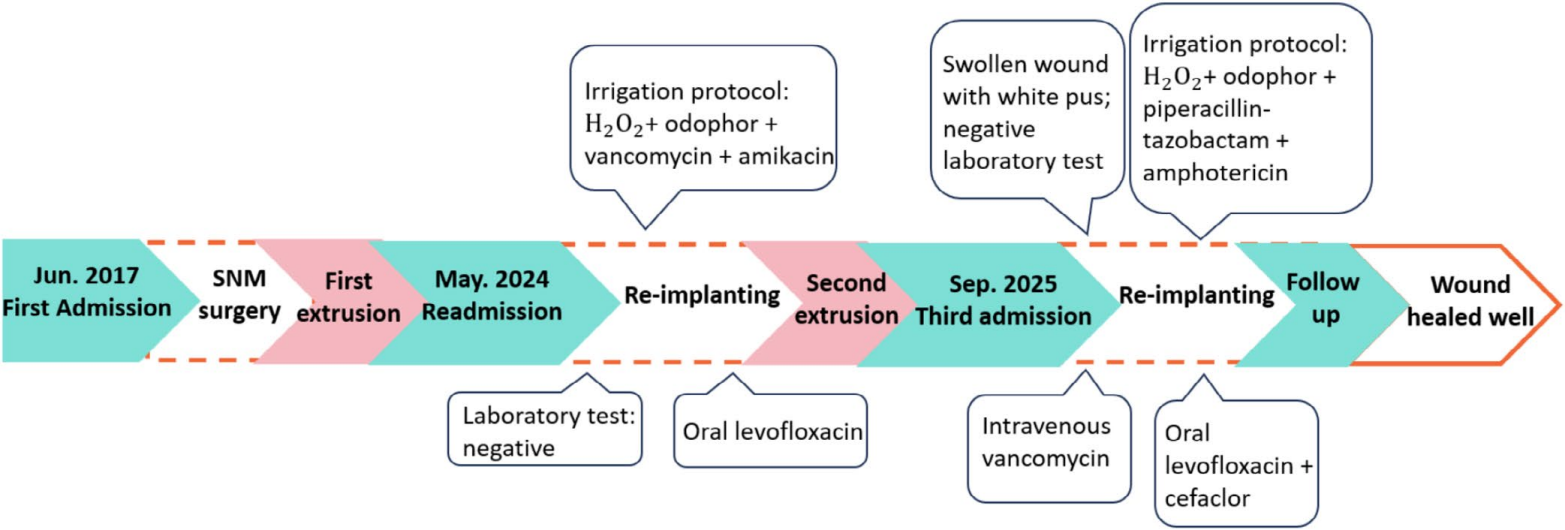
Timeline of the clinical course and management of the patient.

To better illustrate the longitudinal course, a timeline summarizing key events and interventions at each stage is provided in Figure 3.

## 3 Follow‐Up

At the follow‐up visit 1 month later, we found that the patient had followed our instructions and did not continue to engage in heavy physical labor. The wound healed well, without infection or re‐extrusion (Figure 1F). At this stage, we strengthened education focusing on activity limitation and risk‐factor avoidance. He was instructed to avoid heavy lifting, repetitive bending, and to return to work gradually. The patient was also counseled to avoid trauma or pressure over the implant pocket.

At the subsequent 7‐month follow‐up, the patient remained asymptomatic, with effective control of voiding symptoms and no evidence of device‐related complication. Long‐term follow‐up is ongoing.

## 4 Literature Review

We systematically searched PubMed, Scopus, and EMBASE from database inception to December 2024 using combinations of the following keywords: (“sacral neuromodulation” OR “SNM”) AND (“device extrusion” OR “device migration” OR “lead extrusion”). Published case reports describing device‐related complications after SNM were included. We retrieved nine cases of migration or extrusion of SNM implants, including two cases of device skin extrusion; six cases of lead skin migration, exposure, and erosion; and one case of electrode migration. Most of the patients were female (8/9). Among them, reimplantation was conducted in three cases and complete removal in six cases. The median age was 33.5 (interquartile range [IQR]: 26.8, 61.0) years, and the median interval between previous interventions was 7.5 (IQR: 5, 15) months. Table 1 summarizes the clinical information of all patients and Table 2 summarizes the initial management and outcomes of reported cases.

**TABLE 1:** Summary of previously published cases and the present case.

First author	Gender	Principal diagnosis	Age at initial placement	Complication	Interval between previous intervention
Present case	Male	Neurogenic bladder	53	Device skin extrusion	82 months
				Device skin extrusion	16 months
Tariq	Female	Neurogenic bladder	27	Tined lead skin exposure	32 months
Christopher	Female	Urge urinary incontinence	66	Device migration	10 months
				Device skin extrusion	3 months
				Device migration	6 months
				Hemorrhage and pain	5 months
				Recurrence of urinary incontinence	1 month
				Pain	5 months
Ted	Female	Urge urinary incontinence	61	Device skin extrusion	18 months
Tam	Female	Urge urinary incontinence	27	Tined lead protrusion	8 months
Bridget	Female	Overactive bladder	71	Tined lead eroding into the rectum	5 months
Farzaneh	Female	Persistent urinary retention	26	Tined lead eroding into the rectum	15 months
Leonidas	Male	Neurogenic bladder	25	Right‐sided tined lead eroding into the rectum	12 months
Megan	Female	Idiopathic urinary retention	40	Broken tined lead eroding into the sigmoid colon	4 months
Mehdi	Female	Neurogenic bladder	27	Electrode migration	11 months

First author	Management	Trigger	Indication
Present case	Reimplantation	Heavy labor	—
	Reimplantation		
Tariq	Reimplantation	Weight loss	Undergoing sleeve gastrectomy after SNM and lost more than 70 kg
Christopher	Reimplantation	Weight loss	Surgical history: abdominoplasty. Undergoing gastric bypass surgery after SNM and lost 69.8 kg during entire treatment process
	Reimplantation		
	Reimplantation in anterior abdominal wall		
	Removal		
	Reimplantation in the other buttock		
	Removal		
Ted	Removal	Blunt trauma	Medical history: BMI 42, diet‐controlled diabetes
Tam	Reimplantation	Slim build	BMI: 18.4
Bridget	Removal	Spontaneous forward migration of the implanted lead	The patient returned to de novo urinary symptoms
Farzaneh	Removal	Spontaneous migration facilitated by diarrhea	Urinary function recovered after removal
Leonidas	Removal of right‐sided device	Spontaneous migration	Received bilateral SNM implant
Megan	Tined lead removal through sigmoidoscopy	Secondary infection arising from broken lead	Medical history: bipolar depression and asthma. Having received SNM previously and implant was reinserted to the opposite side of buttock 4 months ago, perforation was caused by residual lead
Mehdi	Reimplanating a new electrode	Physiological changes associated with pregnancy and vaginal delivery	Underwent SNM before pregnancy

**TABLE 2:** Management and outcomes of reported cases.

First author	Complication	Initial management	Reimplantation	Reimplantation site	Follow‐up duration	Final outcomes
Present case	Device skin extrusion	Reimplantation	Yes	Same site	4 months	Device remained functional
Tariq	Tined lead skin exposure	Reimplantation	Yes	Same site	6 months	Device remained functional
Christopher	Device skin extrusion	Reimplantation	Yes	Same site	Not reported	Symptom recurred after removal at 6th admission
Ted	Device skin extrusion	Removal	No	—	Not reported	Symptom recurred after removal
Tam	Tined lead protrusion	Reimplantation	Yes	Contralateral buttock	10 months	Device remained functional
Bridget	Tined lead eroding into the rectum	Removal	No	—	3 months	Symptom recurred after removal
Farzaneh	Tined lead eroding into the rectum	Removal	No	—	1.5 months	Urinary function recovered after removal
Leonidas	Right‐sided tined lead eroding into the rectum	Removal of right‐sided device	No	—	3 months	Left‐sided SNM remained functional
Megan	Broken tined lead eroding into the sigmoid colon	Tined lead removal through sigmoidoscopy	No	—	1 week	Perforation resolved after removal; contralateral device remained functional.
Mehdi	Electrode migration	Reimplanating a new electrode	Yes	Same site	3 months	Device remained functional

Nold and McLennon [8] and Roth [9] reported two cases of device extrusion. Extrusion was suspected to have been caused by massive weight loss and blunt trauma, respectively, and the IPG devices were eventually removed. Compared with the patient described in the article by Roth, the patient in Nold and McLennon's report underwent prolonged treatment and was hospitalized six times after the initial implantation 2.5 years prior [8]. Al‐Shaiji [10] reported the case of a 27‐year‐old woman who underwent sleeve gastrectomy after IPG implantation and lost approximately 70 kg, which was considered the main contributor to the extrusion of the tined lead. Since the device responded well and there were no signs of infection, Al‐Shaiji buried the lead [10].

Four cases of lead erosion into the rectum or sigmoid colon were observed. Leonidas et al. reported the complication of rectal perforation in a 25‐year‐old man, who was the youngest among the nine included patients. One year after receiving bilateral full‐system SNM implantation for neurogenic urinary retention, the patient was hospitalized because of tined lead protrusion into the rectum. Subsequently, the right‐sided device was surgically removed, leaving a left‐sided system to treat urinary retention [11]. Sharifiaghdas et al. reported the case of a 26‐year‐old woman who experienced the same complications after SNM. Sharifiaghdas et al. deduced that bowel contraction primarily facilitated spontaneous migration [12]. In the article by Kastelberg et al., the patient revisited the doctor 5 months after SNM, exhibiting lead site drainage, abdominal pain, and hematochezia, and imaging examination confirmed erosion of the lead into the rectum. During surgery, the wound in the rectum was closed by clip under sigmoidoscope [13]. The sigmoid colon perforation in Megan et al.'s report was induced by a lead stub left in situ during the process of changing the previous device. After surgery and antibiotic therapy, the patient was discharged without obvious intra‐abdominal sequelae [14].

Mehdi et al. reported a case of SNM electrode migration found on imaging examination after birth. They suggested that SNM may not affect pregnancy or the fetus; however, physiological and anatomic changes in pregnant women increase the risk of electrode migration. Tam et al. described a rare case of skin protrusions caused by lead. A 27‐year‐old woman with a slim build (165 cm, 50 kg) underwent SNM and 8 months later, she was readmitted to the hospital because of severe pain at the insertion site. They found that the pain was caused by the lead tip penetrating the skin. Therefore, the proximal set of tines was trimmed before reimplantation [15].

When the device or lead was not completely exposed from the skin, Nold and McLennon [7] and Tam attempted a series of salvage reimplantation schemes, but due to repeated extrusion, Nold and McLennon [7] eventually decided to remove it. However, Al‐Shaiji [6] and our case confirmed the intact functionality of the SNM system and planned the reimplantation of devices or wires after adequate anti‐infection treatment. In the four cases in which the lead migrated into the intestinal tract, the outcomes without exception were thorough removal through surgery and endoscopy due to patient discomfort and the potential risk of infection due to lead perforation. When the device failed owing to electrode migration, as in the case described by Mehdi, it was necessary to remove the device. Notably, patient preference for reimplantation was mentioned by Nold and McLennon [7], Al‐Shaiji [10] and in our case as well. This desire might stem from the patient's reluctance to undergo a more complex surgery (complete removal) and concerns regarding cost. Substantial improvement after SNM fortified their intentions with a schedule of salvage reimplantation.

## 5 Discussion

In this article, we present a case of SNM device extrusion caused by heavy labor and summarize nine case reports related to the migration, erosion, and extrusion of SNM implants. Based on our experience with the present case, we emphasize the importance of anti‐infective therapy for the successful management of IPG extrusion. Based on present case and the reviewed literature, risk factors for SNM device migration and extrusion can be broadly grouped into categories: patient‐related factors (e.g., weight loss, labor, and trauma) and device‐related factors (e.g., spontaneous migration and broken leads). From our experience with the present case, we emphasize the importance of anti‐infective therapy for the successful management of IPG extrusion.

**FIGURE 4:**
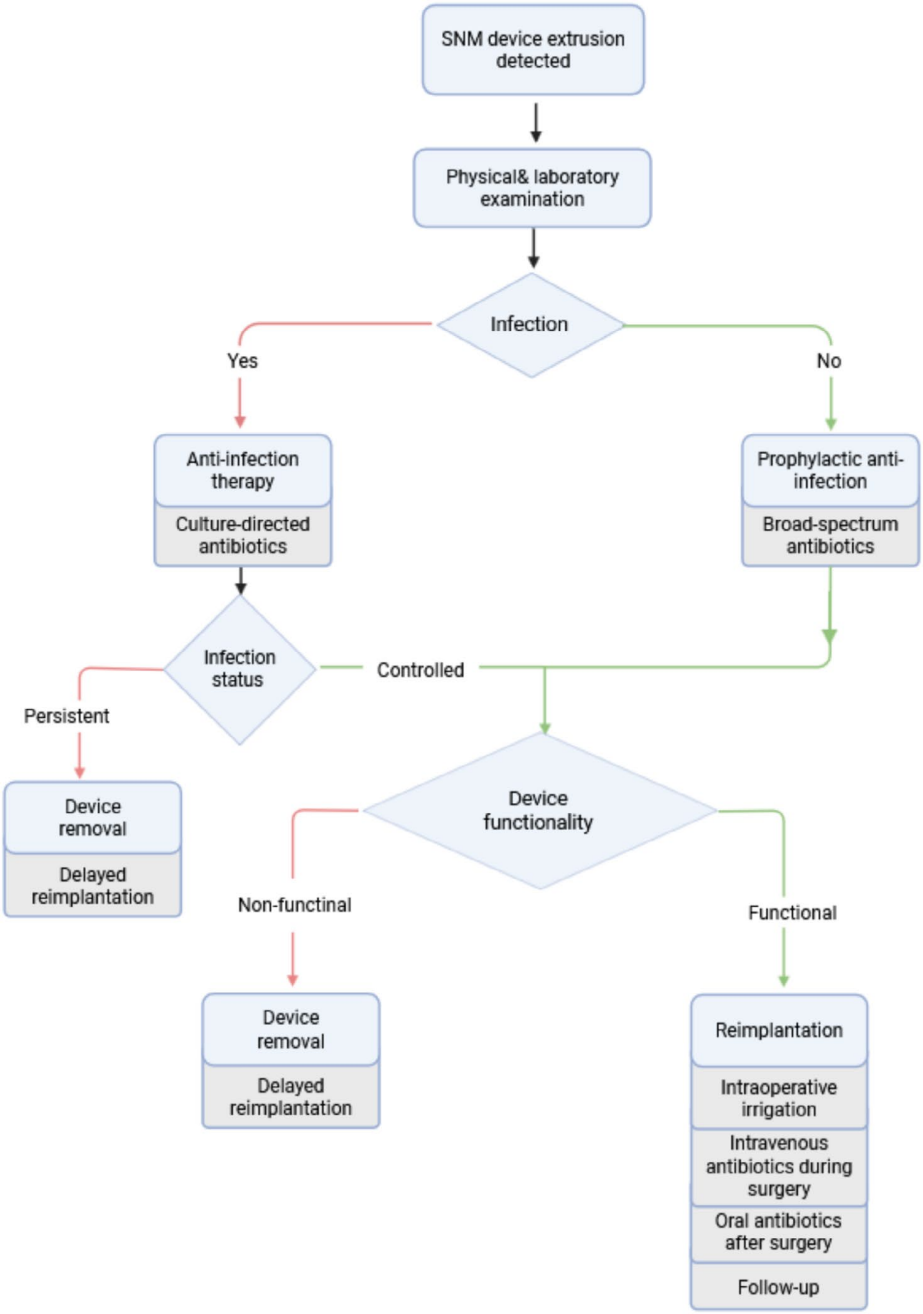
Management algorithm for sacral neuromodulation device extrusion.

Based on our literature review, we found that all the included case reports, except two [9, 15] mentioned the use of antibiotics. The case of Al‐Shaiji, Sharifiaghdas et al. and us introduced a detailed regimen [10, 12]. The above three patients did not have a fever or positive culture results during the perioperative period. Therefore, for patients without obvious infection, these three sets of medication regimens may serve as references. For patients with positive culture findings, culture‐directed antibiotic therapy remains the recommended approach [16]. In our case, in addition to the administration of wide‐spectrum antibiotics, we adopted two irrigation protocols during the operations. The protocol used at readmission, specifically, one‐half strength hydrogen peroxide, one‐half strength iodophor solution, and a mixture of vancomycin and amikacin, was based on the irrigation protocol proposed by Mulcahy [17]. Although the protocol described by Mulcahy was originally designed for infected penile implants, it has been widely used for infections caused by artificial device extrusion in the urinary system [18]. In our case, this protocol mainly targets surface decontamination and common bacterial pathogens in the absence of clinical signs of infection. On the third admission, we changed the irrigation regimen during the operation because of signs of wound infection (swelling and pus). We applied diluted hydrogen peroxide, an iodophor solution, and a mixed solution of vancomycin, piperacillin‐tazobactam, and amphotericin B5, as recommended by Swanton et al. for expanded antibacterial coverage against nontraditional or resistant organisms [19]. In addition, by reflecting on current case and depending on our single‐center experience, surgical site also needs to be considered in the future. Pocket revision at the original site may be suitable for patients with adequate soft‐tissue coverage and limited physical activity, whereas reimplantation at the contralateral buttock offers greater advantages for physically active patients because it avoids previously operated or scarred tissue. To facilitate clinical decision‐making, we designed a management algorithm for SNM device extrusion that integrates infection assessment, evaluation of device functionality, and perioperative management strategies (Figure 4).

In addition, several key points in our case differ from those in the review. First, we present a possibility for device salvage. In cases where the extent of infection is limited and strict anti‐infective protocols (e.g., antibiotic administration and irrigation) have been implemented, migrated or eroded devices can be successfully reimplanted, which should be emphasized in the case. During the third admission, we placed the IPG into a deeper tissue plane to reduce pressure point and mechanical irritation, thereby reducing the risk of device migration or erosion. Furthermore, based on this experience, we underscore the importance of patient education for individuals undergoing similar treatments. Patients should be advised to avoid heavy labor and be fully stressed of the harm associated with prolonged excessive exertion.

According to our literature review, three cases were associated with massive weight loss or low body weight [8, 10, 15], prompting us to reflect on whether a slim body habitus should be considered a potential contradiction. A cohort study revealed no significant difference in complication rates between obese and non‐obese women within 1 year after SNM [20], indicating that the body mass index and body habitus should not be regarded as limiting factors when evaluating eligibility for SMN. However, for patients who have already undergone SNM and intend to lose weight, clinicians should be aware of the risks of device migration and extrusion [6]. We advise clinicians to communicate with patients about any intention or plan to lose weight, and special attention and guidance should be given to patients considering bariatric surgery, as reported in the articles by Al‐Shaiji [10] and Nold and McLennon [8]. Meanwhile, our case highlighted the importance of postoperative patient education in preventing device‐related complications. Patients should be advised to limit physical activity after surgery, such as frequent bending and prolonged squatting. They should also be suggested to regularly check the wound and get medical consultation if they notice pain, redness, or skin changes around the implant site.

Notably, in all five cases of lead migration and erosion, a tined lead was applied, consisting of four units of flexible tines. Once implanted, the flexible tines flare outward within the tissue to secure the lead in the correct position. Although the design of the tined lead primarily prevents outward movement caused by traction, it does not eliminate the risk of anterior migration. A clinical study reported five cases of SNM treatment failure after successful implantation due to lead migration, and anterior migration was noted in four patients (80%) [21]. In some of the case reports identified by the literature review, the outcome was the same: the tined lead in four out of five cases migrated forward and eroded into the rectum or sigmoid colon. Thus, we propose, in a hypothesis‐generating manner, that the invention of a novel bidirectional tined lead may help prevent both anterior migration and outward displacement, thereby potentially improving the stability of SNM therapy. This concept remains speculative and requires further biomechanical validation.

SNM device extrusion is not merely a technical complication, but a multifactorial process driven by device‐related and patient‐specific factors. Careful patient selection, systematic postoperative education, refined device design and follow‐up are essential to prevent recurrence and enable successful device salvage.

## 6 Conclusion

We reported a patient who experienced two episodes of device extrusion due to engaging in heavy labor. A well‐developed anti‐infective strategy, including perioperative antibiotics and irrigation protocols, is an important factor for surgical success. Future study is needed to clarify risk factors and to refine device design to reduce extrusion risk.

## Author Contributions

**Yu‐Sun Guo:** writing – original draft, writing – review and editing. **Wen‐Tong Ji:** writing – original draft, writing – review and editing. **Xiang‐Ze Wang:** data curation, visualization. **Jian‐Long Wang:** conceptualization, funding acquisition, methodology. **Run‐Hua Tang:** formal analysis, methodology.

## Funding

The authors have nothing to report.

## Ethics Statement

The authors have nothing to report.

## Consent

The patient provided written informed consent for the publication of this case report and the accompanying images.

## Conflicts of Interest

The authors declare no conflicts of interest.

## Data Availability

No datasets were generated or analyzed during the current study.
